# Luminal B breast cancer: patterns of recurrence and clinical outcome

**DOI:** 10.18632/oncotarget.11344

**Published:** 2016-08-17

**Authors:** Zhi-hua Li, Ping-hua Hu, Jian-hong Tu, Ni-si Yu

**Affiliations:** ^1^ Prevention and Cure Center of Breast Disease, The Third Hospital of Nanchang City, Key Laboratory Of Breast Diseases In Jiangxi Province, Nanchang, JiangXi 330009, People's Republic of China; ^2^ Department of Pathology, The Third Hospital of Nanchang City, JiangXi Breast Specialist Hospital, Nanchang, JiangXi 330009, People's Republic of China; ^3^ Department of Gynaecology, The Affiliated Hospital of Jiangxi Traditional Chinese Medicine University, Nanchang, Jiangxi 330006, People's Republic of China

**Keywords:** luminal B subtype, breast cancer, recurrence pattern, prognosis

## Abstract

In recent years, most studies on breast cancer relapse and metastasis have focused on non-luminal breast cancers (including the basal-like and HER-2 subtypes) because of their poor prognosis. However, the luminal B subtype is more common, but this type has not been investigated as thoroughly. In the current study, we collected data on 258 patients with luminal-B breast cancer patients with recurrence and metastasis served as the observation group, and 189 patients with non-luminal breast cancer during the same period served as the control group. This study aimed to investigate the pattern of recurrence and clinical outcome after follow-up treatment for luminal B breast cancer. We found a higher proportion of local recurrence and single bone metastasis in patients with luminal B breast cancer than in patients in the non-luminal groups. The risk of recurrence and metastasis in patients with luminal B breast cancer during a 2- to 5-year period and after 5 years was still present, but the risk in patients with non-luminal breast cancers had obviously decreased during the same period. Patients with luminal B breast cancer with recurrence or/and metastasis had a better prognosis after reasonable treatment. The recurrence patterns and clinical outcomes of patients with luminal B breast cancer according to HER2 status were also different, to some degree. These results are of potential clinical relevance especially for the monitoring of clinical prognosis and targeted therapy intervention for luminal B breast cancer.

## INTRODUCTION

Breast cancer is the most common cancer in females worldwide and is the second leading cause of cancer death in women. Despite advances in early detection and comprehensive treatments for breast cancer, approximately 30% of patients with early-stage breast cancer still experience recurrent disease [1]. Most studies [2–4] have indicated that recurrent disease is related to the traditional histopathologic parameters, including lymph node status, histologic grade, and tumor size. The large tumor burdens and micro metastases that are evident *in vivo* prior to relapse are not sensitive to systemic comprehensive treatment and will ultimately lead to cancer recurrence. However, the clinical, pathological and molecular biological characteristics of breast cancers have biologic heterogeneity that these traditional histopathologic parameters fail to characterize. Studies have demonstrated that the intrinsic molecular subtypes can be used to evaluate the distinct features, clinical behaviors and different responses to comprehensive treatment of patients with breast cancer. The different intrinsic molecular subtypes have been defined as follows: basal-like, HER2−enriched, luminal A and luminal B, which each have a different prognosis [5]. Luminal B breast cancers are characterized by a lower expression of estrogen receptor (ER), a low expression of progesterone receptor (PgR) and a high histologic grade [6]. Luminal B breast cancer is defined by aggressive clinical behavior and has a prognosis similar to that of non-luminal cancers (including the HER2−enriched and base-like subtypes) [7]. According to the 2013 St Gallen Consensus, the luminal B subtype accounted for nearly 40% of all breast cancers [8]. Therefore, the pattern of recurrence and clinical outcome of the luminal B subtype should be of concern.

The aim of this study was to analyze the recurrence pattern and evaluate the prognostic features after follow-up treatment in patients with the luminal B subtype of breast cancer.

## RESULTS

### Clinical data of patients who underwent early treatment

The baseline tumor characteristics are listed and compared between the luminal B group and the non-luminal group (Table 1). The median age at diagnosis of the patients with luminal B breast cancer was 48 years (range 31 to 71 years), and the median age at diagnosis of patients with non-luminal breast cancer was 42 (range 28 to 69 years). Compared with non-luminal breast cancer patients with postoperative recurrence and metastasis, the average age at diagnosis of patients in the luminal group was greater, and the proportion of patients with postmenopausal status was higher. The difference between the groups was statistically significant (*P* < 0.005). However, in regards to family history of breast cancer, pathological type, clinical stage, and axillary lymph node status, no differences were observed between the groups. An analysis of individuals with luminal B breast cancer (*n* = 258) showed that patients in this subgroup did not differ in terms of any of the clinical characteristics under investigation (*P* > 0.05).

**Table 1:** Patients' clinical data with early treatments in different subtype breast cancer with recurrence and metastasis

	Luminal B		Non-Luminal	χ^2^	*P* value
	HER-2−	HER-2+			
AGE					
≥ 50	54	65	66	5.644	0.018
< 50	25	114	123		
Menopausal status					
Post-menopausal	53	67	70	4.007	0.045
Pre-menopausal	26	112	119		
Family history of breast cancer					
YES	18	48	37	2.218	0.136
NO	61	131	152		
Histology					
Invasive ductal	72	155	168	0.087	0.768
Others	8	23	21		
Clinical stage TNM stages					
I + II	26	65	78	1.669	0.196
III	53	114	111		
Axillary lymph node status					
No	9	43	52	3.308	0.069
Metastasis	70	136	137		
1–3 node	23	47	48	0.26	0.61
≥ 4 node	57	89	89		
Surgical method					
BCT	8	19	26	1.131	0.288
Mastectomy	71	160	163		
Adjuvant chemotherapy					
CAF regimen	30	48	51	0.154	0.695
CET or AC-T regimen	41	120	115		
No chemotherapy	8	11	13	0.002	0.968
Adjuvant radiotherapy					
YES	58	129	123	2.811	0.094
No	21	50	66		

All patients enrolled in this study were treated according to the guidelines for the clinical diagnosis and treatment of breast cancer. A comparison of patients with luminal B and those with non-luminal breast cancers revealed no difference in these treatments, including surgical procedures, adjuvant chemotherapy and radiotherapy. Moreover, no differences in treatment were found between the HER2+ subgroup and the HER2− subgroup of luminal B patients. In addition, 25 patients with luminal B did not receive endocrine therapy; this was due to a change in ER or PR positivity in 22 cases and to personal reasons in 3 cases.

### Recurrence pattern of luminal B breast cancer

**Figure 1:**
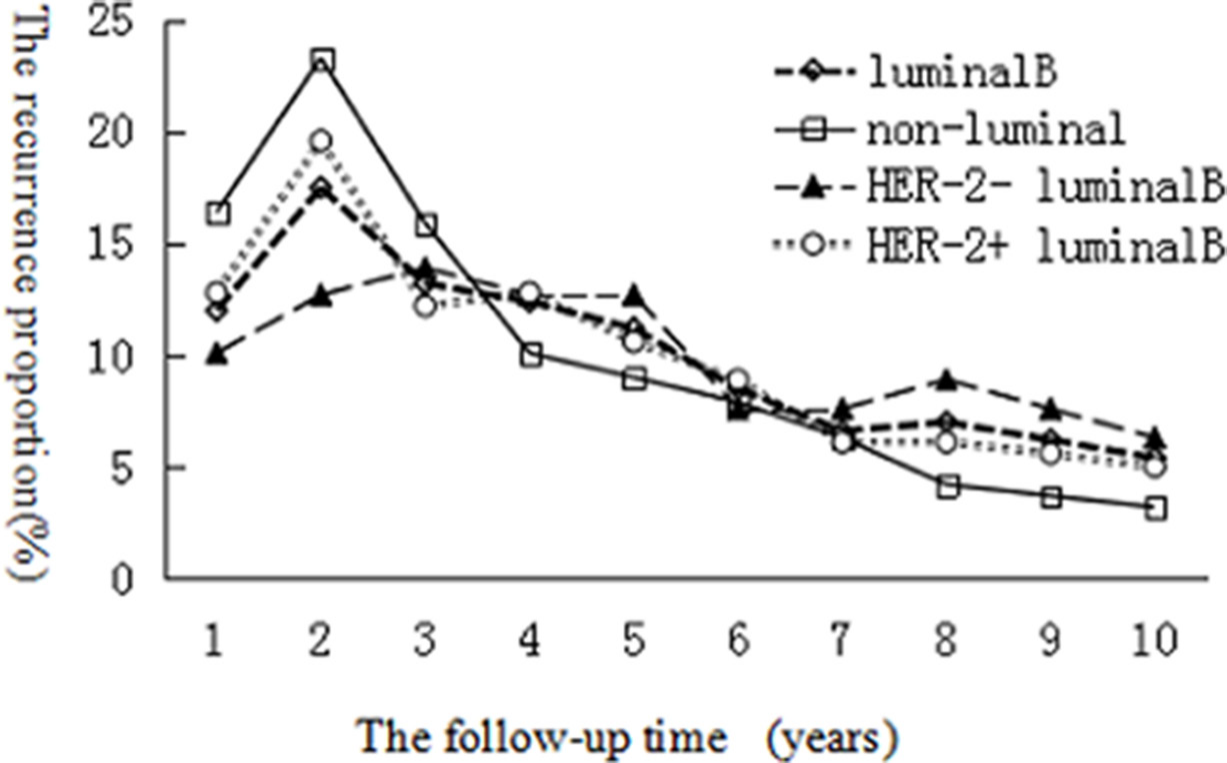
Comparison of BCFI between luminal B and non-luminal breast cancer patients, HER2+ luminal B subgroup and HER2− luminal B subgroup

Among the 258 cases of luminal B breast cancer, 42 patients had local recurrence, 43 cases had regional recurrence, 60 cases had only bone metastasis and 123 cases had metastasis to other sites or multiple organ metastasis. Compared with the 189 patients with non-luminal breast cancer, the proportion of loco regional recurrence and bone-only metastasis was higher in patients with luminal B breast cancer; this difference was significant (*P* = 0.048, 0.038 for non-luminal and luminal B cancers, respectively). Moreover, in luminal B patients, the HER2+ subgroup had a higher proportion of bone-only metastasis than did the HER2− subgroup, and the difference was statistically significant (*P* = 0.023). However, the proportion of loco regional recurrence was not significantly different between the HER2+ subgroup and the HER2− subgroup (*P* = 0.153). The median duration of survival from the time of first disease recurrence was 34, 39, 27 and 22 months for the luminal B group, the HER2+ luminal B subgroup, the HER2− luminal B subgroup and the non-luminal group, respectively. The recurrence risk curves were drawn using the recurrence proportion as longitudinal coordinates and the follow-up time (in years) as horizontal coordinates (Figure 1), and these curves clearly show the breast cancer-free interval (BCFI) of the different groups.

On further analysis, the 2-year cumulative incidence rate was 29.5% (76/258), 30.7% (58/189), 22.8% (18/79), and 39.7% (75/189) for the luminal B group, the HER2+ luminal B subgroup, the HER2− luminal B subgroup and the non-luminal group, respectively. A significant difference was found only between the luminal B group and the non-luminal group (*P* = 0.024). The 5-year cumulative incidence rate was 66.3% (171/258), 68.2% (122/189), 62% (49/49), and 74.1% (141/189) for the luminal B group, the HER2+ luminal B subgroup, the HER2− luminal B subgroup and the non-luminal group, respectively. However, the differences were not statistically significant between the luminal B group and the non-luminal group (*P*= 0.058) or between the HER2+ luminal B subgroup and the HER2− luminal B subgroup (*P* = 0.337). A comparison of the characteristics of recurrence and metastasis between the different groups is shown in Table 2.

**Table 2:** Comparison of recurrence and metastasis characteristics in different group

	Luminal B				Non-Luminal	Luminal B	χ^2^	*P*
	HER2*−*	HER2+	χ^2^	*P*				
Recurrence and metastasis within 2 years								
Yes	18	58	2.44	0.118	75	76	5.099	0.024
no	61	121			114	182		
Recurrence and metastasis within 5 years								
yes	49	122	0.922	0.337	141	171	3.586	0.058
no	30	57			48	87		
Relapse site								
Loco regional recurrence	31	54	2.042	0.153	46	85	3.901	0.048
Distant metastasis	48	125			173	143		
Distant metastasis site								
Only bones	23	37	5.136	0.023	34	60	4.295	0.038
Others	25	88			108	113		

### PFS and MSR of patients with luminal B breast cancer with recurrence and metastasis

Breast cancer patients with recurrence and metastasis were primarily treated according to the current consensus guidelines for advanced breast cancer, personal economic ability, wishes of the patient and the doctor's experience.

**Figure 2:**
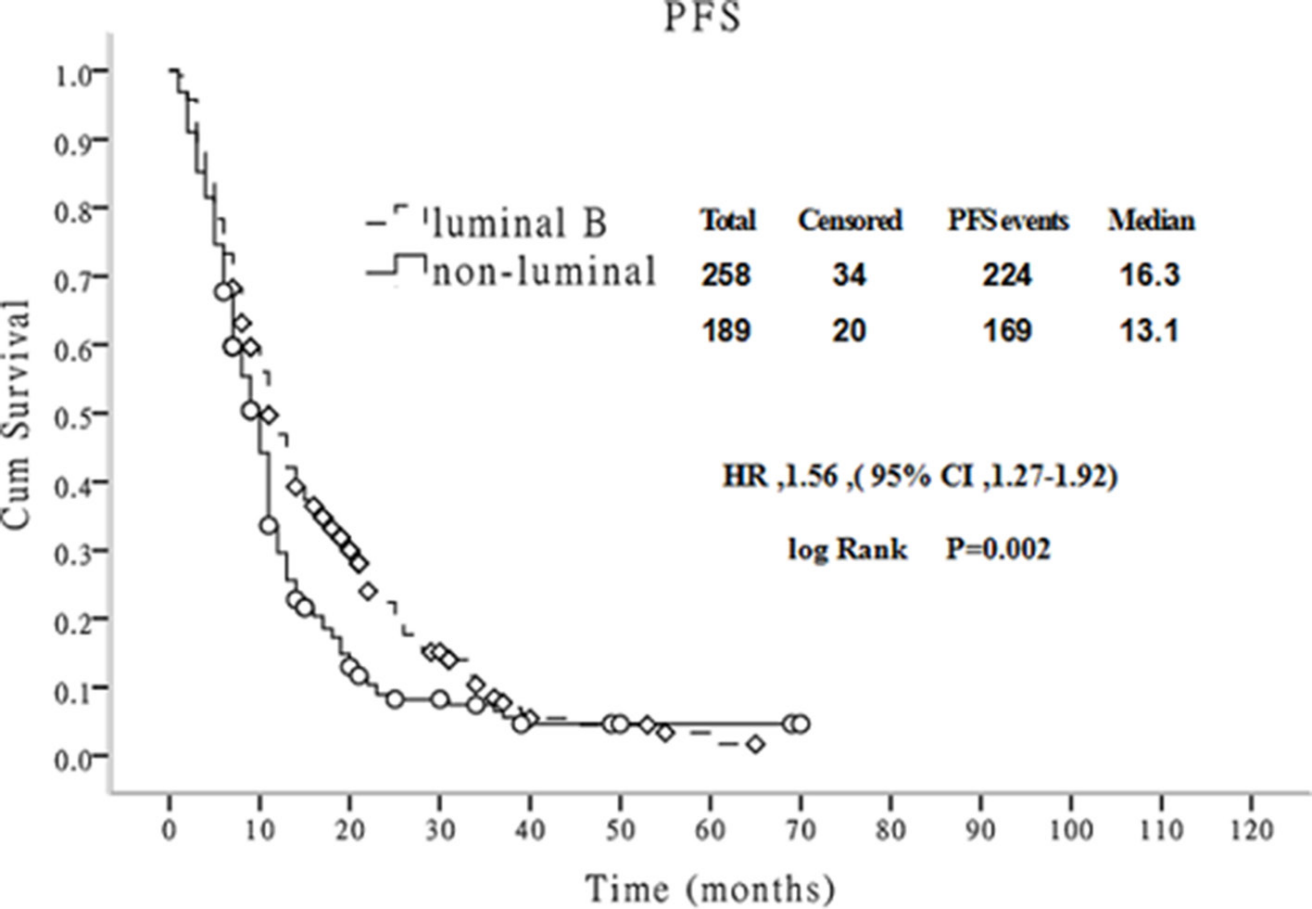
Comparison of PFS between luminal B and non-luminal breast cancer patients

**Figure 3:**
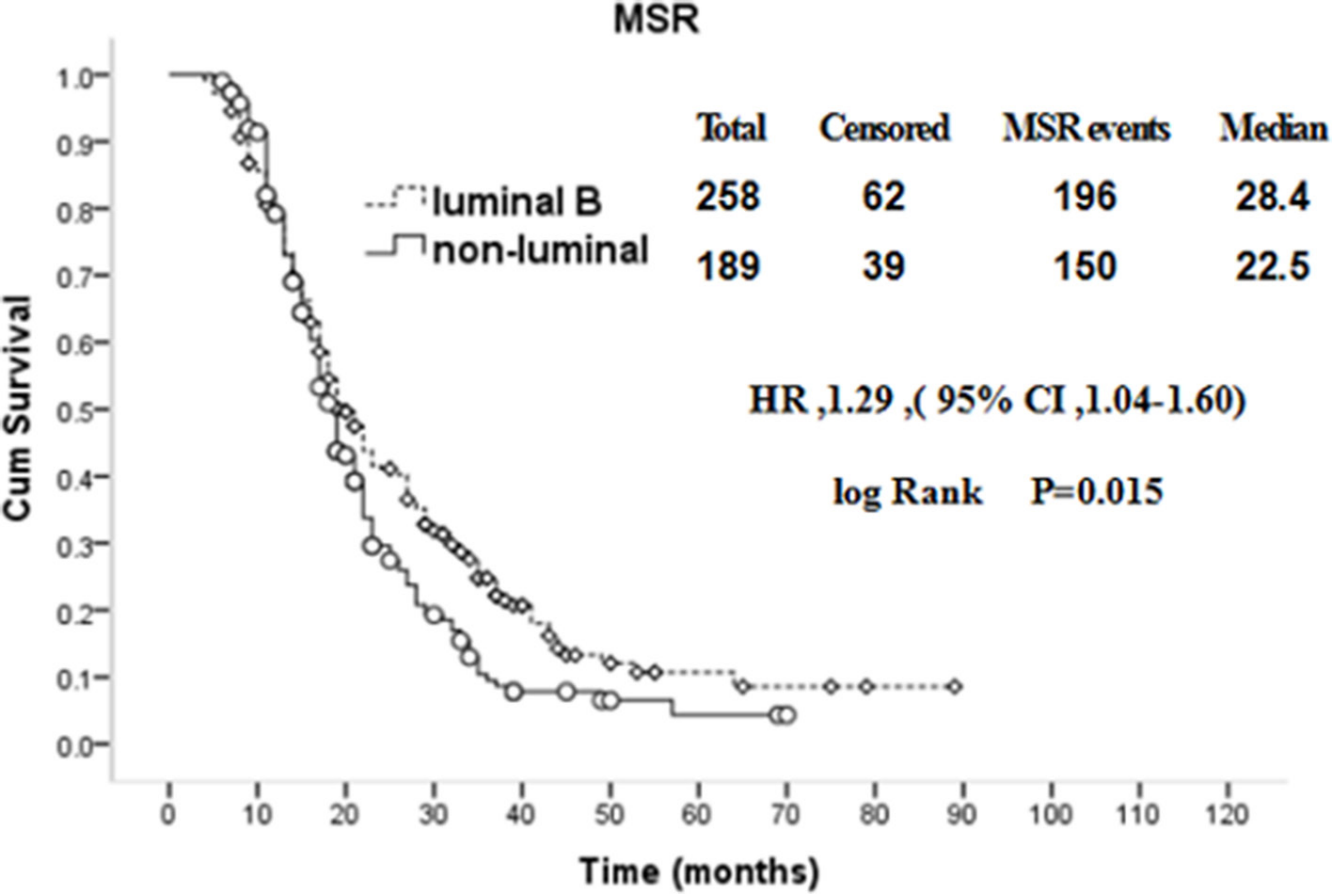
Comparison of MSR between luminal B and non-luminal breast cancer patients with recurrence and metastasis

During the follow-up period, 11 patients in the luminal B group achieved clinical complete response and did not demonstrate progression, but only 2 cases in the non-luminal group did so. The median PFS rates of the luminal B group and the non-luminal group were 20.0 months and 13.11 months, respectively, and this difference was statistically significant (χ^2^ = 9.97, *P* = 0.002) (Figure 2). The median post-metastasis survival time of the luminal B group and the non-luminal group was 28.4 and 22.5 months, respectively. The difference between these groups was statistically significant (χ^2^ = 5.87, *P* = 0.015) (Figure 3).

**Figure 4:**
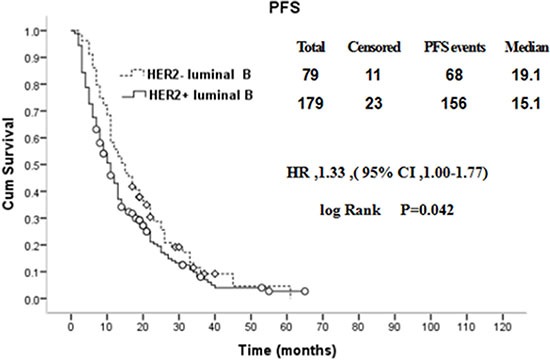
Comparison of PFS between HER2+ and HER2− luminal B breast cancer patients with distant metastasis

**Figure 5:**
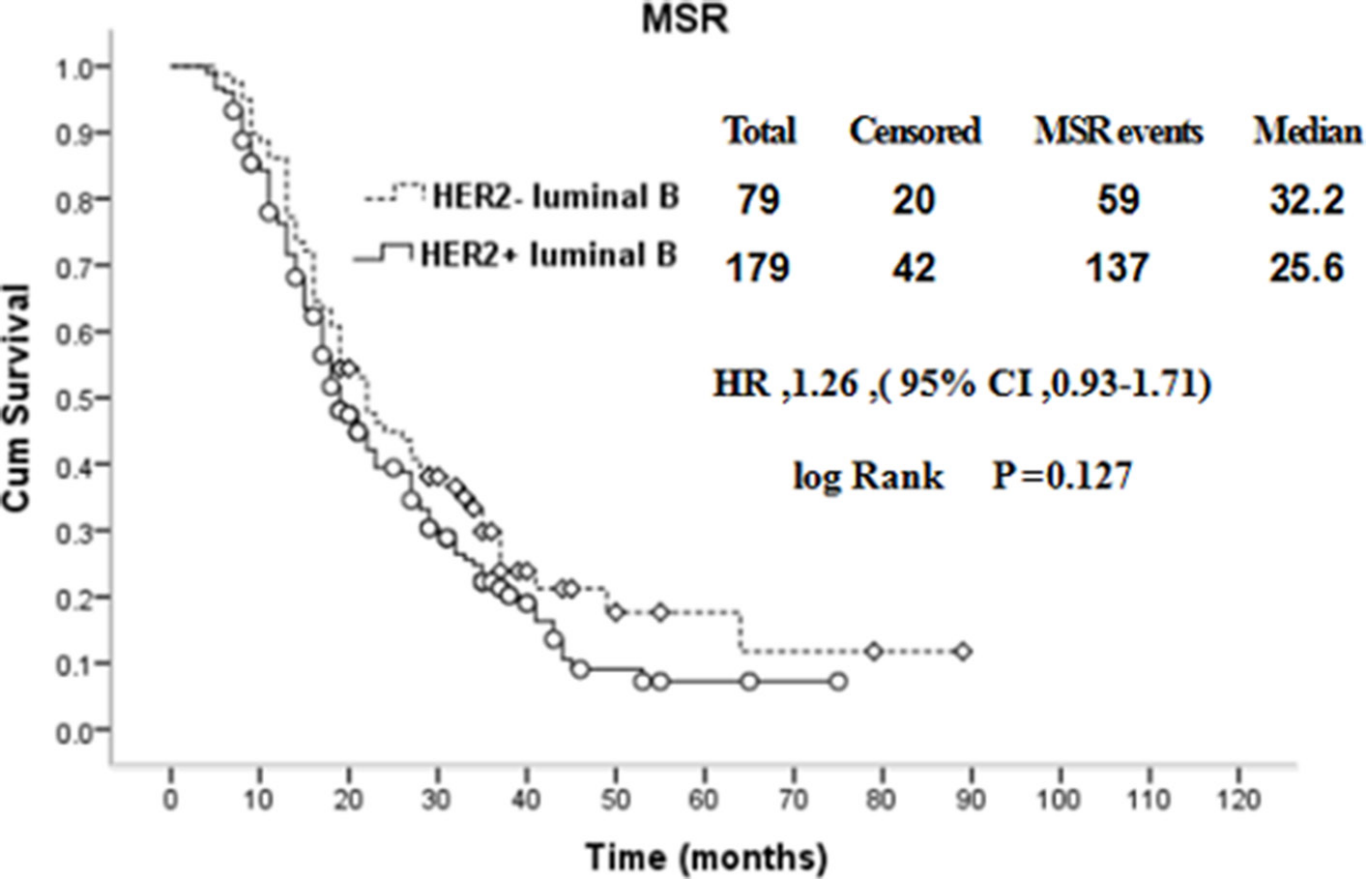
Comparison of MSR between HER2+ and HER2− luminal B breast cancer patients with recurrence and metastasis

The HER-2 gene is an important prognostic indicator for breast cancer patients. In patients with luminal B breast cancer, compared with patients in the HER2+ subgroup, the median PFS of the HER2− subgroup was longer (19.1:15.1 months), and this difference was statistically significant (*P* = 0.042), as shown in Figure 4. In contrast, the difference in the median post-metastasis survival time between the HER2+ subgroup and the HER2− subgroup was not significant (*P* = 0.127), as shown in Figure 5.

## DISCUSSION

The application of gene expression profiling has reshaped our understanding of breast cancer biology. Four main intrinsic molecular subtypes of breast cancer (luminal A, luminal B, HER2−enriched and basal-like) have been classified over the last 15 years, and each of these subtypes has different features, clinical behaviors, and treatment response profiles [9]. Luminal B breast cancer has been reported to have lower expression of hormone receptors, higher expression of proliferation markers, and higher histologic grade than luminal A breast cancer [10]. According to the 2013 St Gallen Consensus, the diagnosis of a portion of patients with the luminal A subtype with poor prognosis was changed to the luminal B subtype, which was determined based on ER positivity, HER2 negativity, Ki67 expression > 14%, and PgR expression < 20% [11–12].

The luminal B subtype is the most common, as this type accounted for approximately 40% of all breast cancers [8]. Remarkably, our study found that 48.1% (258/536) of the patients with recurrence and metastasis had one of the luminal B subtypes. That is to say, compared with the luminal A group, luminal B breast cancer is recognized by a more aggressive clinical behavior and unfavorable prognosis [13]. In the BIG (Breast International Group) 1–98 trial, which assigned 8,010 patients to four treatment arms that compared different sequential administrations of letrozole and tamoxifen, patients with lower ER levels had worse DFS than those with high ER levels [14]. In a meta-analysis of patients with advanced ER-positive breast cancer, HER2 overexpression was identified as risk factor for increased disease recurrence [15]. Most luminal B cancers may have a greater sensitivity to neoadjuvant chemotherapy, but no improvement in disease-free survival was observed in these patients [16]. In ER-positive breast tumors, the loss of PgR or PgR expression < 20% was identified as an adverse prognostic factor [11, 17].

However, the pattern and time to recurrence in patients with luminal B breast cancer were different from those in the non-luminal groups because of endocrine therapy with tamoxifen or AIs. These therapies block the binding of hormone receptors to their corresponding receptors or decrease androgen-derived estrogen formation, which inhibit the proliferation of tumors and reduce the risk of tumor recurrence and metastasis [18–19]. In this study, 258 cases of luminal B breast cancer were compared with 189 cases of non-luminal breast cancer, and it was found that the median age at diagnosis was greater (48:42) and that the proportion of postmenopausal patients was larger (120/258:70/189) in patients with luminal B breast cancer. The difference between the groups was significant (*P* = 0.045). These results are similar to those reported in the literature [20]. After further analysis of the characteristics of recurrence and metastasis in luminal B breast cancer patients, we found that the 2-year cumulative incidence rate and the 5-year cumulative incidence rate were 29.4% (76/258) and 66.3% (171/258), respectively, which were lower than the corresponding rates in non-luminal breast cancer patients. However, the 2–5 year cumulative recurrence rate and the cumulative recurrence rate after 5 years were not lower in the luminal B patients [21]. That is, the risk of recurrence and metastasis in luminal B breast cancer patients from 2 to 5 years and after 5 years was still present, but the risk in the patients with non-luminal breast cancer had obviously decreased during the same period [22]. In a retrospective analysis of breast cancer patients with distant metastasis, women with ER-positive tumors presented a decreased risk of distant recurrence within the first 5 years, but this effect was not seen in ER-negative patients, who showed a decline in risk during the period of 5–10 years after diagnosis [20]. According to the 2013 NCCN guidelines for breast cancer, breast cancer patients who are hormone receptor-positive were recommended to undergo endocrine therapy for at least 5 years after the completion of surgery and chemotherapy [23–24]. However, after 2 years of endocrine therapy, the ER expression pattern varied, and drug-resistance occurred. Features of local recurrence and distant metastasis in patients with luminal B breast cancer that were revealed in this study were consistent with the theory of the guidelines mentioned previously and were confirmed by a number of retrospective studies [20, 25].

The risk of recurrence and pattern of site dissemination in breast cancer depends on factors such as treatment and the intrinsic subtype [26–27]. Local recurrence after radical surgery for breast cancer is typically considered a precursor to distant metastasis. *Engel et al.* [28] found that the risk of distant metastasis in patients with postoperative local recurrence was 3 times higher in patients without postoperative local recurrence. However, some studies found that the postoperative local recurrence of breast cancer was not the only sign of systemic disease. According to prognostic indicators, some patients with local recurrence could be cured [29]. Local recurrence manifests as two different clinical courses. One course is when recurrence occurs many years after surgery, which is usually a slow progression with a relatively better prognosis. The other course is local recurrence that is actually considered a local manifestation of systemic tumor dissemination, which develops quickly and can be thought of as the initial stage of distant metastasis [30]. Breast cancer patients with bone and/or visceral metastases more often have a poor prognosis, but patients with single bone metastasis tend to experience a greater long-term survival. This study found that patients with luminal B breast cancer experienced a higher proportion of local recurrence and single bone metastasis, and had a better prognosis compared with patients with non-luminal breast cancer. Local recurrence and single bone metastasis in luminal B patients may be associated with drug-resistance patterns of endocrine therapy [31–32].

HER2 expression in primary breast cancer has commonly been reported to range from 20 to 30% [33]. HER2 overexpression (2+/3+) was found in 48.57% of the primary lesions and 45.71% of the local-regional recurrences [34]. HER2 positivity was considered an independent prognostic indicator of patient survival and is correlated with a number of adverse prognostic factors in breast cancer including increased occurrence of metastasis and micrometastatic bone marrow disease [35]. *Cheang et al. reported* that the Ki67 index and HER2 status significantly affected the prognosis and clinical outcome of patients with luminal B breast cancer [36]. A comparison of the recurrence and metastasis characteristics between patients with HER2− and HER2+ luminal B breast cancers showed that most cases shared similarities, such as the 2- and 5-year cumulative recurrence rates. In our study, a significant difference was found between the two subgroups with respect to the site of the first distant metastasis. Compared with the HER2+ subgroup, the proportion of patients with bone-only metastasis was higher in the HER2−subgroup (*P* = 0.023). Therefore, according to the 2013 St Gallen Consensus, the portion of patients with the luminal A subtype for which the diagnosis was changed to the luminal B subtype was characterized by ER positivity, HER2 negativity, Ki67 expression > 14%, and PgR expression < 20% [11–12].

The difference in the survival rate of breast cancer with relapse and metastasis was not only related to the subsequent comprehensive treatment, but was also related to the biological characteristics of the tumor itself [21–22, 37–38]. *Lobbezoo et al.* retrospectively analyzed 835 cases of patients diagnosed with metastatic breast cancer from 2007 to 2009, and the MSR were followed-up. Compared with 24.8 months for the HR+/HER2− subtype, 19.8 months for the HR-/HER2+ subtype and 8.8 months for the TN subtype, the longest survival was observed for the HR+/HER2+ subtype (median 34.4 months) (*P* < 0.0001) [39]. In our study, patients with non-luminal breast cancer with relapse and metastasis had a poorer prognosis than patients in the luminal B groups, which was the case for both the PFS and MSR. Interestingly, the PFS of patients with HER2− luminal B breast cancer was better than that of patients with HER2+ luminal B breast cancer, but the MSR was not significantly different. The cross talk between the HER2 and ER signaling pathways in breast cancer contributes to resistance to hormonal therapy. The combination of trastuzumab and anastrozole produced statistically significant improvements in PFS, TTP, CBR, and ORR in postmenopausal women with HER2+ luminal B MBC [40]. In or study, some patients with HER2+ luminal B MBC received trastuzumab therapy, which affected the PFS and MSR. This result suggests that if we are to make an impact in terms of a decrease in the mortality of early breast cancer, we should focus on the search for additional therapies for the different subgroups of luminal B disease.

This study has several limitations. First, it was a retrospective analysis with a small sample size. Second, the guidelines for the diagnosis and treatment are constantly updated, the economic situation of the patients and patient perception in regards to treatment often change, the experience of the doctors accumulates and the treatment of patients with metastasis and recurrence is different (e.g., more HER2 + patients have received HER2−targeted therapy in recent years); all of these may lead to differences in the outcome. Third, in all patients with recurrence and metastasis, the luminal B subtypes accounted for a much higher proportion than that which has been reported recently [8, 41]. In addition, there may have been potential selection/information and confounding bias.

In conclusion, a higher proportion of local recurrence and single bone metastasis was observed in patients with luminal B breast cancer compared with patients with non-luminal breast cancer. The risk of recurrence and metastasis in luminal B breast cancer patients during a 2- to 5-year period and after 5 years was still present, but the risk in non-luminal patients had obviously decreased during the same period. Luminal B breast cancer patients with recurrence or/and metastasis had better prognosis after reasonable treatment. The recurrence patterns and clinical outcome of luminal B breast cancer patients according to HER2 status were also somewhat different, which indicated that precise individual therapy might contribute to an improvement in clinical outcome.

## MATERIALS AND METHODS

### Patients

In all, 536 patients with breast cancer with recurrence and metastasis after their first surgery were treated at the Third Hospital of Nanchang City from January 2005 to 2015 June. These cases accounted for 4.7% of the hospitalized breast cancer patients during this same period. In all, 258 patients with luminal B breast cancer and 189 patients with non-luminal breast cancer were enrolled in this study. All tissue sections were subjected to immunohistochemistry (IHC), and the results were reviewed by a consultant breast cancer pathologist (Jian-hong Tu) for histological classification and immunohistochemical assessment. The patients were diagnosed with either luminal B or non-luminal breast cancer (including the HER2−enriched and basal-like subtypes) according to the National Cancer Institute guidelines. Positive ER and PR status was determined when immunostaining was positive in ≥ 1% of the cells [42]. HER2−positive cancers were defined by either strong membrane staining (3+) observed by IHC or amplification of HER2 confirmed by fluorescence *in situ* hybridization when immunohistochemistry detected moderate (2+) membrane staining [43].

According to the HER2 status, the patients with luminal B cancer were stratified into two groups as follows: the HER2+ group and the HER2− group. The differences in the recurrence patterns and clinical outcomes were evaluated between luminal B and non-luminal breast cancer patients and were evaluated in the HER2+ and HER2− subgroups of luminal B patients. This study was approved by the ethics committee of the Third Hospital of Nanchang City, and written informed consent was obtained from all patients.

### Follow-up and endpoints

The cut-off for the follow-up period was December 31, 2015. The site of recurrence was classified as a local (ipsilateral breast or chest wall, including mastectomy scars), regional (ipsilateral axillary, infraclavicular, internal mammary, or supraclavicular), or distant metastasis (bone marrow, lung, liver, brain and other organs). Overall survival (OS) was calculated from the time of breast cancer (BC) diagnosis to the time of death or the last follow-up. Disease-free survival (DFS) was defined as the time from the diagnosis of BC to the first local, regional or distant recurrence. Progression-free survival (PFS) was defined as the interval from the start of treatment to disease progression or time of death. Post-metastasis survival (MSR) was defined as the interval from recurrence and metastasis at any site to death by any cause or to the time of the last follow-up.

### Statistics

All statistical analysis was performed with the SPSS statistics program version 19.0 (SPSS, Inc., Chicago, IL, USA). The Chi-square test was used to compare the baseline tumor characteristics, such as age, menopausal status, family history of breast cancer, histological cancer type, clinical stage, axillary lymph nodal status, surgical technique used, random assignment to a chemotherapy-containing regimen, the site of initial recurrence and the initial time to recurrence. PFS and MSR curves were calculated using the Kaplan-Meier method, and the differences between the luminal group and the non-luminal group were compared using the log-rank test. A *p*-value < 0.05 was considered statistically significant.

## Abbreviations

ER: estrogen receptor
PR: progesterone receptor
HER: human epidermal growth factor receptor
IHC: immunohistochemistry
BC: breast cancer
FISH: fluorescence in situ hybridization
CET: cyclophosphamide epirubicin and taxane
AC-T: cyclophosphamide and epirubicin-taxanes
CAF: cyclophosphamide epirubicin and 5-Fu
HR: hormone receptor.
PFS: progression-free survival rate.
MSR: post metastasis survival rate.
